# Classification of childhood obesity using longitudinal clinical body mass index and its validation

**DOI:** 10.21203/rs.3.rs-5392188/v1

**Published:** 2024-12-16

**Authors:** Vidhu Thaker, Nia Ebrahim, Apurva Khadegi, Shuliang Deng, Kun Qian, Zonghui Yao, Shaleen Thaker, Benjamin May, Nandan Patibandala, Sara Lopez-Pintado

**Affiliations:** Columbia University Irving Medical Center; Columbia University; Columbia University; Beth Israel Deaconess Medical Center; Mailman School of Public Health; NorthEastern University; Columbia University Irving Medical Center; Columbia University Irving Medical Center; Boston Childrens Hospital; NorthEastern University

**Keywords:** childhood obesity, body mass index, cardiometabolic risk

## Abstract

**Objective:**

Persistence of childhood adiposity is known to be associated with long-term adverse cardiometabolic risks. Yet, cross-sectional body mass index (BMI) is often used to classify obesity in clinical care and research. This study aimed to develop and validate a childhood obesity classification system using longitudinal clinical data.

**Methods:**

This observational study used electronic health record data from a tertiary care hospital, with replication in an independent cohort. For individuals with ≥ 3 BMI measurements, the median BMI percentile and persistence in the obesity class were used for longitudinal classification. The association between longitudinal BMI class and early onset obesity, socioeconomic status (SES), and adverse cardiometabolic risk was analyzed.

**Results:**

Height, weight, and cardiometabolic risk measures were obtained for 22 352 children from 2014–18 in the primary cohort and 24 444 children in the replication cohort. Obesity (BMI ≥ 95^th^ percentile [BMI_pct95_]) was observed in 24.1% and severe obesity (BMI ≥ 120% of BMI_pct95_) in 10.6%. Individuals with early onset obesity (age ≤ 6 years) remained in the same or higher obesity class; those in the high-SES group had lower odds of obesity. The proportion of individuals with cardiometabolic risk increased with increasing obesity severity (p-value 0.01 - < 0.001). The AUC for cardiometabolic risk by longitudinal BMI class was higher compared to that for cross-sectional BMI (80.0% vs. 75.8%, p < 0.001).

**Conclusions:**

The Longitudinal BMI classification may better reflect long-term cardiometabolic risk in children. This classification can be useful for focused intervention strategies and for profiling of individuals for genetic testing.

## Introduction

The rising trend of obesity, especially severe obesity, in children and youth in the US^1^ is a risk to the cardiometabolic health of the nation. In 2022, the Centers for Disease Control published new pediatric growth charts with extended body mass index (BMI) for age to accommodate BMI up to 60 kg/m^2^ which have been widely integrated into many electronic health record (EHRs) systems for clinical care. Obesity was defined as BMI for age and sex ≥ 95^th^ percentile and severe obesity as ≥ 120% of 95^th^ percentile (%BMI_95pct_)^2^. To align with the adult definition of obesity, Class 1 obesity is considered age- and sex-specific BMI > 95^th^ to < 120% BMI_95pct_, Class 2 obesity as BMI ^3^120% of BMI_95pct_ or ^3^35 kg/m^2^, whichever is lower, and Class 3 obesity as BMI ^3^140% of BMI_95pct_ or ^3^40 kg/m^2^. This classification allows uniformity across the lifespan of clinical care, population-based studies, and other research. It also allows for clinical tracking of BMI in children and youth when BMI values are beyond the plotting axes on population-based growth charts, especially in those with severe obesity.^3^

Longitudinal studies have shown that childhood adiposity is associated with adverse cardiometabolic risk.^4–6^ Individuals with consistently high adiposity from early age to adulthood are at increased risk, while those with a reduction in adiposity reverted to the same risk as individuals without obesity.^4^ This observation raises an important question about using cross-sectional data, especially in children, where changes in the level of obesity as measured by BMI are well-known as they progress through different phases of growth.^7,8^ Longitudinal trends and their association with cardiometabolic risk have frequently been assessed using latent class generalized linear mixed models^5,9^ or mixed effect models.^6^ These models are useful for examining the trends of BMI trajectories within the study cohort, but less so for the clinician caring for a patient at the point of care. Finally, the availability of data from EHRs provides a valuable resource for research. However, the errors in such datasets preclude the use of a single measurement to define a condition, and the aggregation of repeated measures is a way to overcome this limitation. The goal of this study was to create a framework for clinicians and researchers to classify obesity using longitudinal BMI measurements collected in a clinical context and to assess their association with cardiometabolic risks using data from clinical health records and validation in an independent dataset from another clinical center.

## Methods

### Data Sources

#### a. Demographic and Clinical Data

This study included subjects ages 2–20 years attending the primary care and weight management outpatient clinics at Boston Children’s Hospital (BCH), a free-standing tertiary care children’s hospital, from 2014 to 2019 as the discovery dataset, and from the Pediatric Primary Care Clinics at Columbia University Irving Medical Center (CUIMC) as the replication dataset. Demographic data, including race/ethnicity, primary language, health insurance, street address, and zip codes, were extracted. Health insurance was assigned to public, private, or other categories based on a reference library maintained by the Clinical Research Informatics groups at the respective hospitals. Lifetime data on height/weight, blood pressure, and laboratory test results pertinent to cardiometabolic outcomes (lipid panel, hemoglobin A1c (HbA1c), alanine transferase (ALT), and ICD-codes) were extracted from the clinical data warehouse of the EHR at (Cerner Powerchart, Kansas City, MO, USA) and CUIMC (Eclipsys, Atlanta, GA, USA).

#### b. Socioeconomic Indicators

Data on socioeconomic indicators including population, food insecurity estimates, unemployment rate, poverty rate, median income, population distribution by race/ethnicity and home ownership by census tract as well as county level data on the meal gap, the food budget shortfall and count of the persons living above and below the 200% of the federal poverty level was provided by the Greater Boston Food Bank (GBFB).^10^ The census tract for the study subjects was obtained using the street address and zip code using the US Census Bureau geocoder (https://geocoding.geo.census.gov/geocoder/).

### Anthropometric measurements and assignment of BMI class

Height, weight, and blood pressure were measured during routine clinical care by trained medical assistants for individuals aged 2–20 years of age. BMI was defined as the weight (kg)/height (m) squared from the primary recorded anthropometric data. Biologically implausible values (BIV) of the anthropometric parameters were removed. The external criteria for BIV were height z-score < −5 or > 4 and BMI z-score < −4 based on the data from population-based CDC 2000 growth charts.^11,12^ Any extreme measurements within subject BMI, BMI-z, and absolute differences in velocity were removed using mixed-effect models.^13–15^ Individuals with at least three BMI readings measured over a minimum of 6 months were selected for longitudinal BMI classification. The SAS program for the 2000 CDC Growth charts^16^ was used to obtain percentiles and z-scores for anthropometric measurements. Gender-specific BMI class was based on the 2022 CDC extended BMI-for-age growth charts as follows: a) normal BMI between 5 –84^th^ percentile; b) overweight when BMI was between 85–94^th^ percentile; c) Class 1 obesity for BMI between 95^th^ percentile −120% of BMI_95pct_; d) Class 2 obesity for BMI between 120–140% of BMI_95pct_; and e) Class 3 obesity for BMI > 140% of BMI_95pct_. To define the longitudinal BMI category, the median BMI percentile of the individual measurements for each participant was obtained. The final class assignment was made when > 50% of the BMI readings were in the specific class and the median BMI percentile for the subject was in the same category. For ties between the two criteria, the lower BMI class was assigned. A manual review of 200 random cases was performed by two authors to confirm the class assignment with 97% concordance. Early onset obesity (EOO) was defined in a similar way using BMI measurements for individuals aged ≤ 6 years.

### Clustering of socio-economic indicators:

The subjects were clustered into three levels of socioeconomic status (SES) using k-means clustering from census tract-level data on food insecurity, unemployment rate, poverty rate, median income, and home ownership. The clustering efficiency was checked by inertia, defined as the ratio of the within-cluster sum of squares to the total sum of squares.

#### Early onset obesity:

The BMI readings for age ≤ 6 years were subset and classified into categories of normal, overweight, Class 1–3 obesity based on the BMI definition for age- and sex-specific percentiles, similar to that for the longitudinal BMI classification.

### Laboratory measurements and definition of adverse cardiometabolic risk factors:

Laboratory measurements were performed in the clinical laboratory at BCH and CUIMC as part of the clinical care. Subjects with Type 1 diabetes and cystic fibrosis were removed using ICD codes. In the clinical laboratory, the test assays were performed on Roche/Hitachi Cobas^®^ system analyzer using the following methods: 1) triglycerides: lipoprotein lipase enzymatic test (CV range 0.7–2.0%); 2) high-density lipoprotein cholesterol (HDL-C) and total cholesterol: enzymatic colorimetric method (CV range: HDL-C: 0.5–1.5%; total cholesterol: 0.6–1.6%); 3) low-density lipoprotein cholesterol (LDL-C): Friedewald calculation (TC = HDL-C+LDL-C+VLDL-C, where VLDL-C is defined as TG/5 in the fasting state); 4) glucose: hexokinase enzymatic test (CV range 0.5–1.3%); 5) insulin: electrochemiluminescence immunoassay (ECLIA, CV range 1.1–4.9%); 6) hemoglobin A1c: electrochemiluminescence immunoassay (CV range 0.5 – 2%); 7) alanine transferase: spectrophotometric assay (CV range 0.6 – 3.5%).

The cardiometabolic risk factors were defined as follows: a) total cholesterol ≥ 170 mg/dL, HDL-C < 40 mg/dL, LDL-C ≥ 130 mg/dL, VLDL-C > 30 mg/dL, non-HDL-C ≥ 145 mg/dL, and triglycerides ≥ 100 mg/dL for children 2–9 years and ≥ 130 mg/dL for children > 9 years of age;^17^ b) hemoglobin A1c ≥ 5.7%;^18^ c) ALT levels as a measure for hepatic steatosis (50 U/L for boys and 44 U/L for girls).^19^ SBP/DBP was classified as abnormal if the measurement was ^3^ 95^th^ percentile for age, sex, and height based on the reference tables for blood pressure as defined by the AAP Clinical Practice guideline on the management of high blood pressure in children and adolescents.^20^ BP was classified into categories: normal, elevated, Stage 1, and Stage 2 hypertension as defined in the AAP guideline^20^. When multiple laboratory results or BP measurements were available, the last available value was selected for analysis. The study was approved by the institutional review boards (s) of BCH and CUIMC.

### Validation of BMI Categories:

Validation of the assigned BMI categories was performed using the known associations of childhood obesity: a) the association of early onset obesity with that in later ages, b) the association of cardiometabolic risk factors with higher levels of obesity, and c) the association of higher obesity with lower SES.

## Statistical Analysis

The smoothed median longitudinal BMI from each class was used to visualize the BMI class compared with the CDC 2000 growth charts and 2022 extended BMI growth charts. A functional boxplot is represented for each BMI class, showing differences in the shapes of the BMI trajectories across groups. To assess the presence of rebound adiposity in each BMI class, the derivatives of the smoothed BMI curves were calculated, and the first time point at which the derivative was zero was considered as the time of adiposity rebound. The distribution of these time points was compared between the classes. Multinomial regression was used to identify the association of the longitudinal obesity class (outcome) with socioeconomic status or EOO as the predictor while adjusting for significant covariates of sex, race/ethnicity, and insurance status. The probability of longitudinal BMI class with the predictor of interest (SES or EOO category) was calculated. The presence of cardiometabolic risk factors by BMI class was assessed by bivariate analysis and compared using the chi-square test. A chi-square test for trend was performed to assess the higher cardiometabolic risk with increasing BMI class. To understand the association of BMI class with the presence of cardiometabolic risk factors, multivariable analyses using generalized linear models with a logarithmic link were used, while adjusting for age, sex, race, and ethnicity. The exponents of the coefficients were used to report odds ratios with confidence intervals, with the BMI category of 5–95^th^ percentile as the reference. To compare the classification based on the longitudinal versus cross-sectional BMI classes, similar associations were made using the first-available BMI for the subjects and a randomly selected BMI value. Similar methods were used for the BMI class and EOO assignment in the replication cohort. The association of BMI class with EOO and cardiometabolic risk factors was examined in the replication cohort. Statistical significance was set at p < 0.05. All statistical analyses were performed using SAS software (version 9.3, SAS Institute Inc., Cary, NC, USA) and R statistical software (version 4.3.2)^23^.

## Results

**Figure 1:**
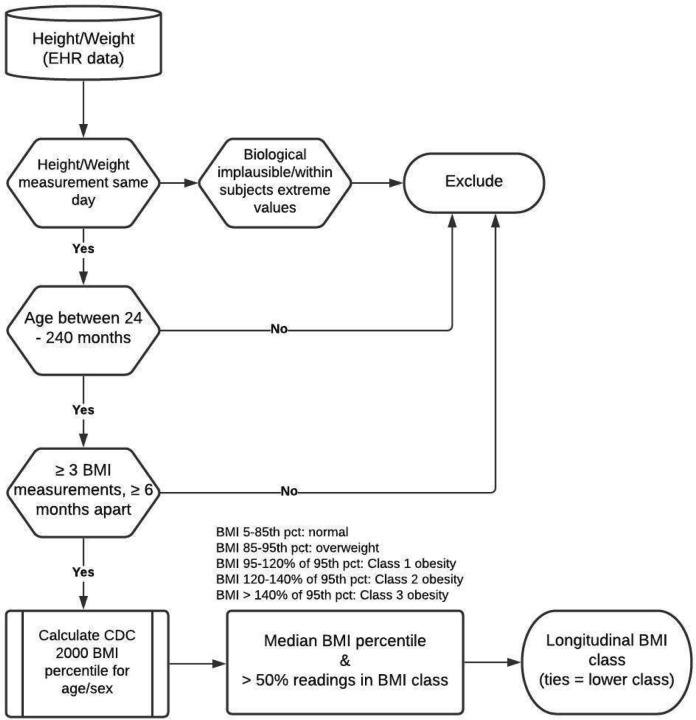
Workflow for the longitudinal BMI classification using repeated measures of BMI obtained during clinical care.

**Figure 2:**
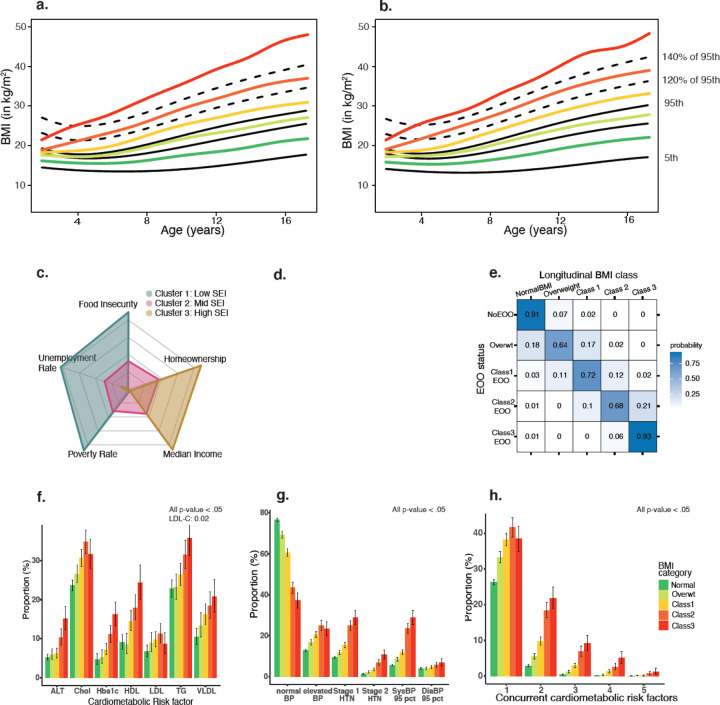
Longitudinal BMI category and its validation. Median BMI from the longitudinal BMI classification plotted on the CDC 2000 reference charts a) for males, b) for females; c) Spider plot of 3 clusters identified by k-mean clustering for 5 variables pertinent to of socio-economic status (SES) with clear separation; d) heatmap showing the probability of being in the longitudinal BMI class predicted by the class of early onset obesity (EOO) while adjusting for race and insurance status; e) proportion of subjects with each cardiometabolic risk factor based on the longitudinal BMI category; f) proportion of subjects with different levels of blood pressure by longitudinal BMI category; g) presence of concurrent cardiometabolic risk factors based on the longitudinal BMI category.

The dataset extracted from the BCH data warehouse included 32 076 unique children attending 4 outpatient clinics (Primary care, Weight management, Endocrine and Bariatric Surgery program). A total of 484 564 BMI measurements were obtained from lifetime clinical visits to the medical system, where both height and weight were obtained simultaneously. Height and weight readings under two years (n=108 173 observations, 22.3%) were removed. A total of 2.4% of the readings were removed as errors: biologically implausible values for height (height z-score < −5 or >+4, n=2 865), BMI (BMI z < −4, n=402), and within subjects’ extreme values (change in BMI, BMIz, and velocity, n= 5 983). A total of 5 796 unique subjects were excluded for < 3 BMI readings over a minimum of 6 months (Supplementary Table 1). The final dataset consisted of a total of 355 579 BMI observations of 22 353 unique individuals, 50.2% female, representing 125 464-person years was used for further analyses (median BMI readings 10, IQR = 13, range 3– 265). Based on the algorithm used for classification of BMI categories (Figure 1), 59.8% (n = 13 374) children had normal BMI, 16.1% (n = 3 600) were overweight, 14.1 % (n = 3 128) were in Class 1, 6.4% (n = 1 432) in Class 2, and 3.7% (n = 818) in Class 3 obesity. The specific distribution of each obesity class according to race is shown in Table 1. There was an approximately equal distribution by sex in the overall study sample and each subcategory in the analysis. The median BMI of each category conforming to the classification system is shown by sex in Figure 2 a & b. The replication dataset was obtained from the Pediatric Primary Care clinic at CUIMC, located in Washington Heights, NY. The dataset was filtered using similar methods, and the final cohort included 174 729 observations of 24 444 unique individuals (Supplementary Tables 1 and 2) representing 95 143 person-years.

**Figure 3:**
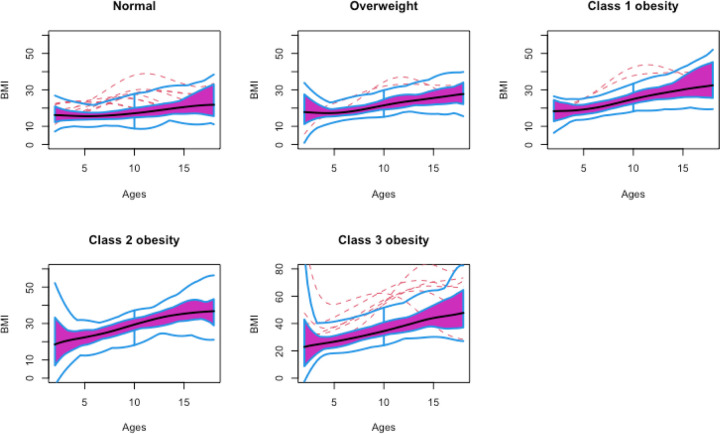
Functional boxplots for each BMI trajectory class. The median curve is represented with a dark line in the middle of the 50% central region. Obesity rebound is seen in the first three groups (normal BMI, overweight and class 1 obesity), but not in the severe obesity (BMI ≥ 120% of BMI_95pct_). The dashed lines represent outliers.

The adiposity rebound time was detected by our statistical approach in 97% of the children in the normal weight group, 68.7% in the overweight group, and in 43.6%, 7.7%, and 13.6% of children in the three classes of obesity, respectively. This shows that adiposity rebound is easily identified in the normal group and uncommon in children with obesity. The functional boxplots^21,22^ in Figure 3 show the medians and 50% central regions in each group to describe the distribution of the curves and detect outliers. The distribution and shapes of the BMI curves were different across groups, and the rebound only appeared clearly in the normal and overweight groups, but not in any group with obesity.

### Socio-economic indicators:

The data on socio-economic indicators from the GBFB were linked to 18 242 subjects from the study cohort. K-means clustering was used to group subjects with similar socioeconomic status using the five available variables–food insecurity, unemployment rate, poverty rate, median income, and home ownership–into three predefined clusters to represent low, mid, and high SES (Supplementary Table 3). The total within-cluster sum of squares was 28.8% of total sum of squares, indicating a reasonable separation of the groups (Figure 2 c). Compared to the reference group with low SES, there were lower odds of Class 1 obesity (OR 0.82, 95% CI 0.73–0.92, p <.001), Class 2 obesity (OR 0.81, 95% CI 0.69–0.95, p =.01), and Class 3 obesity (OR 0.67, 95% CI 0.53–0.83, p <.001, Supplementary Table 4).

### Early onset obesity:

A total of 12 322 subjects had data on BMI measurements available at age ≤ 6 years and were assigned early onset BMI classes, such as those for longitudinal BMI. Ninety-one percent of individuals with normal BMI at age ≤ 6 years continued to have the same longitudinal BMI class. Among the children with early onset obesity, 85–93% continued to remain in the same class of obesity or higher with the longitudinal BMI class. The subjects had a high probability of remaining in their respective BMI classes or higher across the different classes of early onset obesity, while adjusting for gender, race, and insurance status (Figure 2 e and Supplement Table 5).

### Cardiometabolic risk factors:

A total of 170 117 laboratory readings were obtained for 14,663 children, and 274 293 BP readings for 22 829 unique children (SBP = 274 281, DBP = 273,516) were obtained. After removing the observations for children with type 1 diabetes and cystic fibrosis (n = 6 170), the last available laboratory results (35 975 observations of 12,112 individuals) and BP measurement (32 590 observations of 19 478 individuals) were used as the outcome measures. The median age for the measurement of the laboratory tests was 11.6 years (IQR 7.5), while the last available blood pressure reading was 9.8 years (IQR 6.9). The proportion of individuals with abnormal laboratory test results using the pre-specified reference ranges was higher with increasing obesity class according to longitudinal BMI, and the trend was statistically significant for each of the laboratory measures as well as the blood pressure measurements (Table 2 and Figure 2, f–g).

There were higher odds of cardiometabolic risk factors with increasing severity of obesity compared to children with BMI between 5–95^th^ percentile for age in both the primary study cohort (Table 3) and the replication cohort (Supplementary Table 6), while adjusting for age, gender, race, and ethnicity. Finally, we tested for the co-occurrence of cardiometabolic risk in the same individual(s). The prevalence of multiple cardiometabolic risk factors increased significantly with an increase in the severity of obesity (Figure 2 h). For the sensitivity analysis, we assessed the association of ≥ 2 cardiometabolic risk factors with the obesity class assigned by the first available BMI reading or randomly assigned BMI reading while adjusting for age, sex, and race/ethnicity. The AUC for the longitudinal BMI class was higher compared to the class assigned by first available BMI measurement (80.0% vs. 75.8%, p <.001), or randomly selected BMI measurement (80.0% vs. 77.0%, p=.002). Similar results were obtained in the replication cohort (73.6% vs. 69.6%; p <.001).

## Discussion

In this study, we devised a novel and versatile method to classify childhood obesity using longitudinal BMI measurements collected during routine clinical care based on the percent of BMI_95pct_ of the CDC 2000 clinical growth charts. This method is easy to use and leverages persistence in certain classes of obesity. It was conceived and validated in the primary dataset and replicated well in an independent clinical dataset. The data collected in clinical care in EHRs provides opportunities for clinical care, clinical research, and population health studies. Height and weight measures used to calculate BMI are frequently recorded (96.5% and 48.8% of the time for weight and height, respectively),^23^ but either may not be measured according to protocol,^24^ or may be prone to transcriptional and conversion errors within the EHR systems.^23,25^ Hence, use of a single data point of BMI to classify obesity is fraught with imprecision. Pediatric providers often see children over a period, have access to repeated measures for the same individual, and can easily overcome the limitations of cross-sectional data. Longitudinal studies following children into adulthood, such as the Bogalusa study^26^ or the Cardiovascular Risk in Young Finns Study^27^ have shown that persistence of adiposity from childhood to adulthood is closely associated with a higher risk of cardiovascular risks such as carotid intima thickness and various other measures of subclinical atherosclerosis measured in the third decade of life or later.^26–29^ Some studies have also shown resolution of cardiometabolic risk in individuals who have been able to change the level of adiposity from childhood to adulthood.^4,30^ While this is encouraging, there is high persistence of obesity in individuals from early life to adulthood,^31^ also noted in our study, where the majority of children in a given BMI category under 6 years of age either remained in the assigned obesity category or moved to a higher one.

Many studies have used longitudinal data to classify obesity and identify its associations with long-term cardiometabolic consequences.^5,6,9,32–34^ Most of these methods use proprietary statistical software, can be applied only to the cohort of subjects under study, and are difficult to extend to a new patient in the clinic. Furthermore, the ability to integrate the system into EHRs will facilitate its wide application and access for pediatricians. We extended our previously published algorithm designed to identify severe obesity using EHR data from three large tertiary care hospitals,^35^ to develop a method that can be used by clinicians at the point of care. Freedman *et al* have shown the inaccuracy of the BMI z-score as an appropriate measure to gauge the severity of obesity in two studies of longitudinal BMI measurements.^36,37^ Hence, we used the most recent system of percent of BMI_95pct_ for this algorithm. The classification system was validated by the known persistence of early onset obesity later in life and the association of cardiometabolic risk factors in the discovery dataset and replicated in an independent dataset. Furthermore, the prevalence of obesity was mapped to the social factors in the discovery set. Cross-sectional studies of nationally representative datasets of children,^38,39^ the prevalence of cardiometabolic risk factors increases with higher degrees of obesity measured by BMI. Aris *et al* have shown the relevance of early age at BMI rebound using the longitudinal trends of BMI as a strong predictor of adverse cardiometabolic risk profiles in adolescence, emphasizing the use of longitudinal BMI measurements collected in routine clinical care for targeted preventive interventions^9^.

In this study population, there was no difference in the trends of obesity between sexes, as has been shown by prior national trends.^40^ The prevalence of class 1, 2, and 3 obesity was similar to that of national trends from the most recent NHANES data.^41^ However, the difference in prevalence of obesity in African American and Latino children compared to the children self-reported Whites was not as pronounced as that seen from nationally representative data. Similarly, while the prevalence of severe obesity was lower in self-reported Asian children, there was no significant difference between self-reported White, African American or Latino ancestry. This likely reflects the fact that children of all races with higher degrees of obesity are more likely to be seen at a tertiary care center than in the community.

We observed higher odds of each cardiometabolic risk factor when compared with children with a BMI between 5–95^th^ percentile. As observed in a cross-sectional study of NHANES data by Skinner *et al,*^38^ this relationship was less strongly observed with LDL and not with Class 3 obesity. However, in contrast to this study, we observed higher odds for all the remaining cardiometabolic risk factors, emphasizing the relevance of using the longitudinal BMI classification. We also reviewed the association of non-HDL-C in this cohort, as non-HDL-C is considered better than other lipid measures in predicting adult dyslipidemia and subclinical atherosclerosis.^42^ However, as both VLDL-C and non-HDL-C are derived measures, these were not used in assessing the presence of multiple risk factors. The Bogalusa study was the first systematic assessment of the presence of multiple cardiometabolic risk factors with increasing BMI.^43^ This finding has been replicated in larger studies since then.^29,44,45^ This study also observed a linear trend in the presence of multiple adverse cardiometabolic risk factors in the same individual, a grave reminder to all pediatric providers and the families they serve about the oncoming epidemic of cardiometabolic diseases in future generations.

This study was undertaken at a tertiary care center and replicated in a primary care facility of a medical center in the Northeast US. These two locations serve demographically different populations, validating the application of the method, particularly with large sample sizes. The use of data collected in clinical care enhances the applicability of the method in non-research settings, particularly without bias for voluntary participation. However, it is limited by sparse data for both BMI and cardiometabolic outcomes. The trends in both obesity prevalence and cardiometabolic outcomes matched nationally representative samples; hence, we have confidence in its wide applicability. Because the study sample was drawn from large medical centers, it included children with a wide range of diseases. To overcome this limitation, we excluded children with a known diagnosis of cystic fibrosis or type 1 diabetes. In addition, the large population with BMI between the 5–85^th^ percentile provides a suitable reference group to assess the impact of the higher classes of obesity on the cardiometabolic risk profile. With the wide availability of EHR data, future studies should address these issues using data drawn from community-based practices from a larger geographical distribution. Future studies could also use this classification to assess the association of genetic, socioeconomic, behavioral, or other similar factors that have been shown to be either associated with or causative of childhood obesity.

## Conclusion

This study utilized clinical EHR-based data to develop a classification system for childhood obesity in a discovery dataset that was mapped to known associations with cardiometabolic risk, early onset obesity, and social factors. This obesity classification system is superior to using random cross-sectional measurements. It can be easily incorporated into EHRs for better guidance for clinicians, profiling for genetic testing, and for higher vigilance and care for long-term metabolic risk.

## Supplementary Material

Supplement 1 Tables 1 to 3 are available in the Supplementary Files section.

## Data Availability

The datasets generated during and/or analysed during the current study are not publicly available due to privacy restrictions by the respective institutional review boards. These data can be made available from the corresponding author on reasonable request and with appropriate data use agreements.

## Data Sharing:

Data described in the manuscript, code book, and analytical code are available upon request with appropriate institutional data-sharing agreements.
