# Supplementary material for: Classification of childhood obesity using longitudinal clinical body mass index and its validation
Source: Res Sq. 2024 Dec 16:rs.3.rs-5392188. Preprint. [Version 1] doi: 10.21203/rs.3.rs-5392188/v1 (PMC11702781; doi:10.21203/rs.3.rs-5392188/v1)
Supplement: Supplement 1 — Tables 1 to 3 are available in the Supplementary Files section. [file NIHPPRS5392188v1-supplement-1.pdf]

Supplementary Files

This is a list of supplementary files associated with this preprint. Click to download.

- Supplementtables.pdf
- Table1.docx
- Table2.docx
- Table3.docx
